# Deployment of aortoiliac balloon-expandable covered stent via the transradial approach: report of two cases

**DOI:** 10.1186/s42155-026-00655-1

**Published:** 2026-01-26

**Authors:** Yasuyuki Tsuchida, Naoki Hayakawa, Toshiki Tsurumaki, Hiromi Miwa, Masanao Inoue, Shinya Ichihara, Shunichi Kushida

**Affiliations:** Department of Cardiovascular Medicine, Asahi General Hospital, I-1326, Asahi, Chiba 289-2511 Japan

**Keywords:** Endovascular therapy, Transradial approach, Aortoiliac disease, Covered stent

## Abstract

**Background:**

Endovascular therapy (EVT) for aortoiliac (AI) disease has shown favorable outcomes with recent advances in technology. Furthermore, the transradial approach (TRA) has emerged as a less invasive alternative to transfemoral access, improving patient comfort. However, data on CS implantation using the TRA remain limited.

**Case presentation:**

We report two cases in which a VIABAHN® VBX-CS (W.L. Gore & Associates, Flagstaff, AZ, USA) were successfully deployed for AI disease using the TRA. In both cases, a 6-Fr transradial guiding sheath was used. The CSs were advanced and accurately deployed using a TRA without complications. The completion of the angiography confirmed the proper expansion and positioning of the CS. Both patients were discharged uneventfully without access-site complications.

**Discussion:**

These cases demonstrate the feasibility and safety of AI balloon-expandable CS implantation using the TRA. The evolution of the device has enabled the delivery of relatively large CS using the TRA, expanding the applicability of this less invasive technique.

**Conclusion:**

Transradial CS treatment may be a promising procedure for AI lesions, offering both procedural safety and patient comfort.

## Introduction

Endovascular therapy (EVT) in the aortoiliac (AI) lesion has demonstrated excellent results with the use of bare-nitinol stents (BNS) and advanced technology [1–3]. However, in severely calcified or complex lesions, BNS alone may be insufficient, and covered stents (CS) have increasingly been utilized in recent years [4–7]. Although CS implantation has demonstrated favorable results in the AI region [8–12], recent progress in access strategies has also influenced EVT practice.

With ongoing device evolution and the miniaturization of delivery systems, the feasibility of performing EVT for AI lesions via the transradial approach (TRA) has been established [13, 14]. TRA provides several procedural advantages, including reduced access-site complications, shorter hemostasis time, and earlier ambulation, which can contribute to improved patient comfort and perioperative management, as shown in the COMFORT study [15].

Moreover, newer balloon-expandable CS, such as the BXB (GORE® VIABAHN® VBX covered stent with the low-profile VBX delivery system, W.L. Gore & Associates, Flagstaff, AZ, USA), are now compatible with 6-Fr systems through radial access. In this paper, we report two cases in which BXBs were safely and accurately deployed in AI lesions by the TRA.

## Case report

### Case 1

**Fig. 1:**
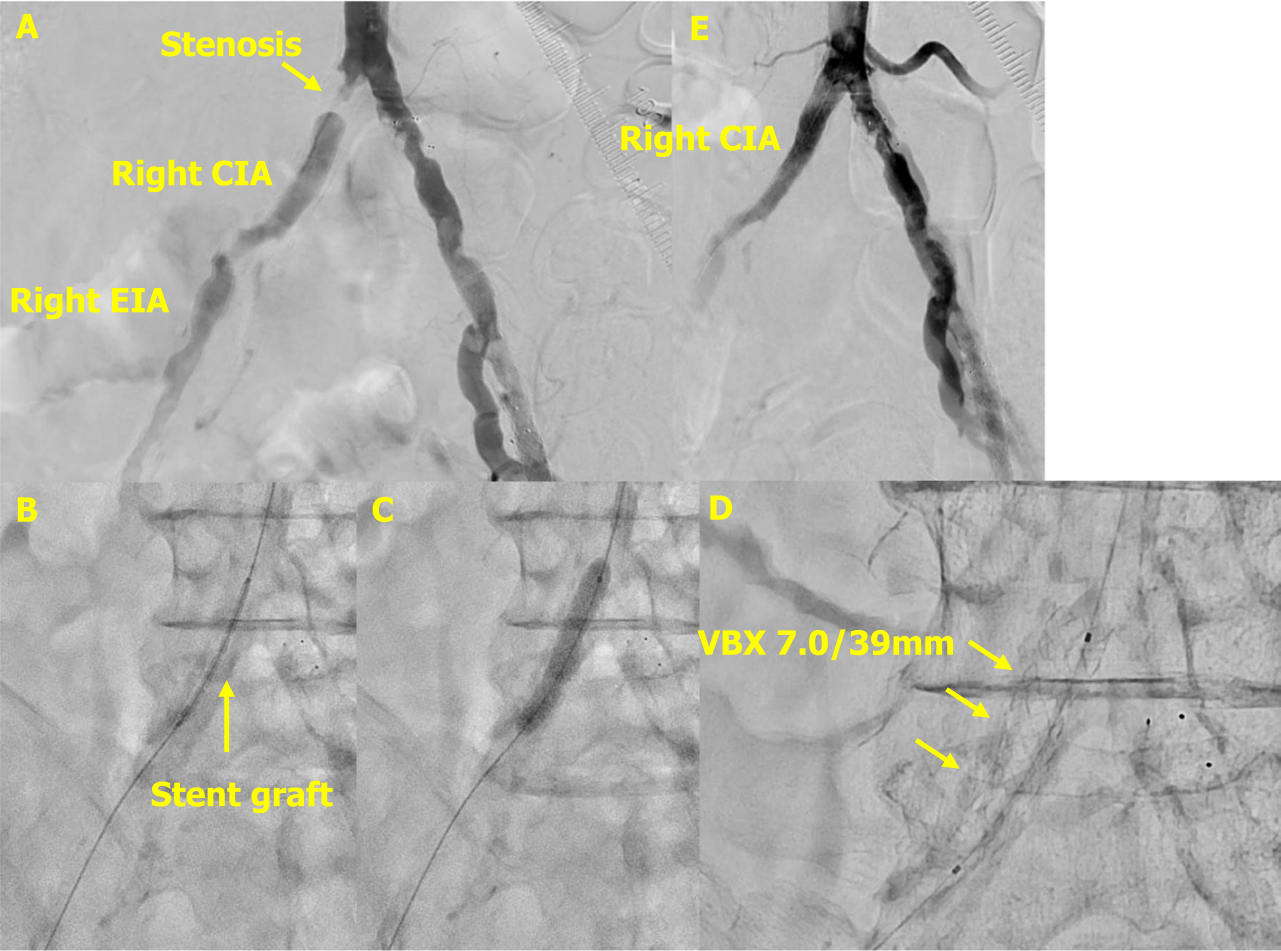
Case 1: Endovascular therapy for stenosis of the right common iliac artery. **A** Stenosis was observed in the right common iliac artery. **B** The covered graft was successfully delivered using the transradial approach. **C** The VBX 7.0/39 mm was deployed and expanded at the ostial lesion of the right common iliac artery. **D** The covered stent was confirmed to be successfully deployed in the right common iliac artery. **E** Final angiography demonstrated satisfactory expansion of the right common iliac artery

A 78-year-old man with a history of diabetes mellitus and peripheral arterial disease had previously undergone stent implantation from the left common iliac artery to the left superficial femoral artery. He presented with several months of intermittent claudication of the right lower limb, for which EVT was performed. A 6-Fr Glidesheath (Terumo Corp., Tokyo, Japan) was inserted through the left radial artery, and a 4-Fr JR 4.0 catheter was advanced along with a Radifocus guidewire (Terumo Corp.) through the aortic arch into the terminal aorta. Angiography confirmed severe stenosis of the right common iliac artery (CIA) with calcification (Fig. 1A), the system was exchanged for a Destination Slender (Terumo Corp.), and a Gladius MG ES wire (ASAHI INTECC Corp., Aichi, Japan) was advanced through the lesion, followed by pre-dilatation with a 5-mm balloon. Subsequently, a VBX 7.0/39 mm (W.L. Gore & Associates, Flagstaff, AZ, USA) was deployed by the TRA (Fig. 1B, C, D). Post-dilatation with an 8-mm balloon was performed, and final angiography confirmed favorable results (Fig. 1E). The procedure was completed without puncture-site complications, the patient was able to walk immediately after returning to the ward, and an improvement in claudication of the right lower extremity was confirmed.

### Case 2

**Fig. 2:**
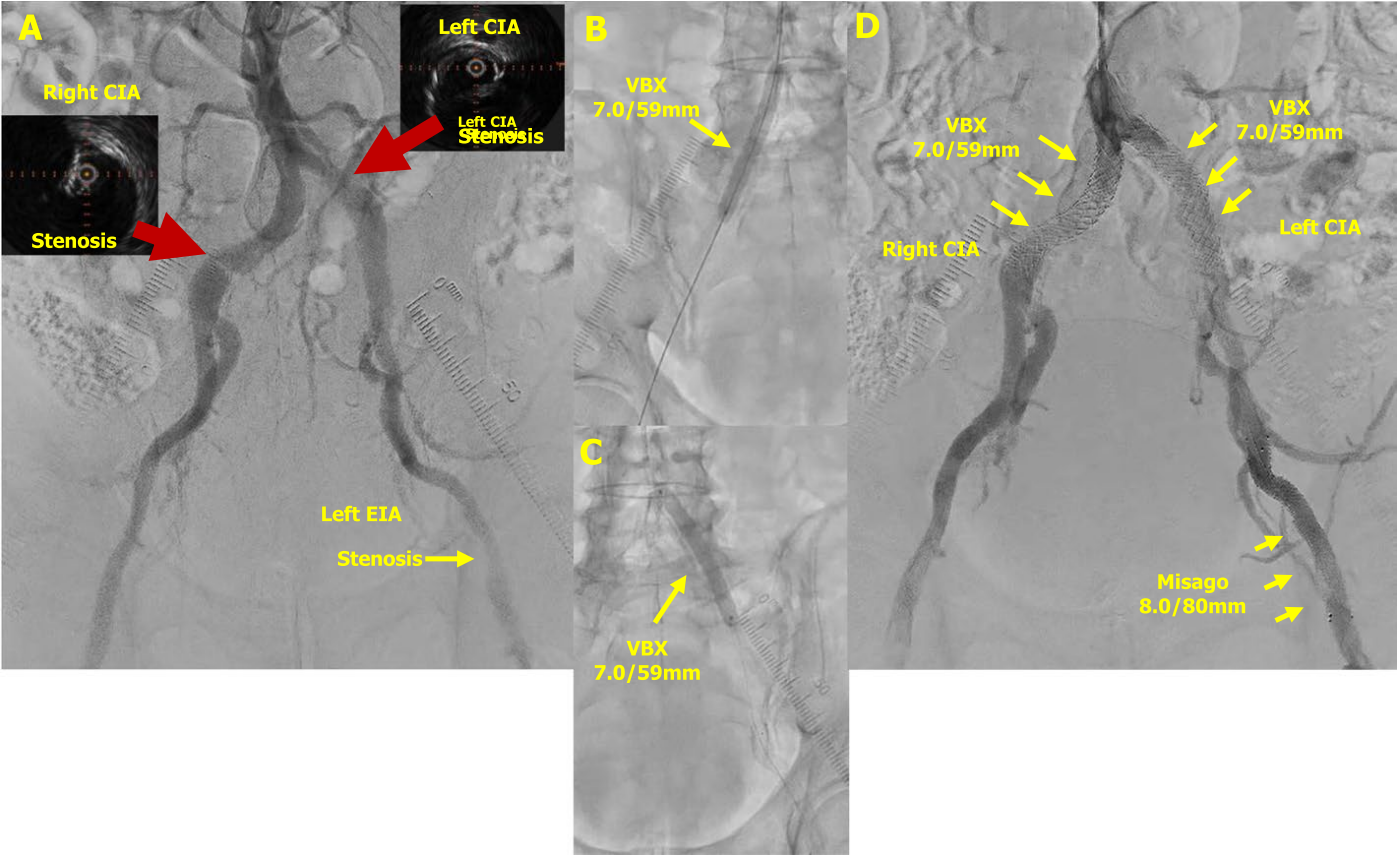
Case 2: Endovascular therapy for stenosis of the right common iliac artery. **A** Stenosis was observed in both common iliac artery and the left external iliac artery. **B** The VBX 7.0/59 mm was deployed and expanded at the proximal lesion of the right common iliac artery. **C** The VBX 7.0/59 mm and the Misago 8.0/80 mm stent were deployed at the ostial lesion of the left common iliac artery and the left external iliac artery, respectively. **D** Final angiography demonstrated satisfactory expansion of both common iliac artery and the left external iliac artery

A 76-year-old man with a history of hypertension and diabetes mellitus presented with several months of intermittent claudication of both lower extremities. The ankle-brachial index (ABI) values were 0.71 on the right side and 0.68 on the left side. Therefore, an EVT was performed. A 6-Fr Glide Sheath was inserted through the left radial artery, and a 4-Fr Pigtail catheter with a Radifocus guidewire was advanced across the aortic arch to the terminal aorta. Angiography confirmed severe stenosis of both CIA and left external iliac artery (EIA) with calcification (Fig. 2A), and the system was exchanged for a Destination Slender. After advancing the Gladius MG ES wire across the right-sided lesion, the lesion was evaluated by intravascular ultrasound (IVUS). Severe stenosis caused by calcified plaque was identified in the right CIA. After pre-dilatation with a 7-mm balloon. Subsequently, a VBX 7.0/59 mm was deployed in the right CIA (Fig. 2B). Post-dilatation with a 9-mm balloon was performed. Similarly, the guidewire was advanced across the lesion on the left side, and the lesion morphology was evaluated using IVUS. Severe stenosis due to fibrous plaque was observed in the left EIA, and severe stenosis caused by calcified plaque was noted in the left CIA. Therefore, a Misago 8.0 × 80 mm stent (Terumo Corp.), which is a BNS, was deployed in the left EIA, and a VBX 7.0 × 59 mm was implanted in the left CIA (Fig. 2C). Post-dilatation of the left CIA was performed with a 9-mm balloon, and final angiography confirmed favorable results (Fig. 2D). The procedure was completed without puncture-site complications, and the patient was able to walk immediately after returning to the ward. Improvement in claudication of both lower extremities was confirmed at outpatient follow-up.

## Discussion

These cases highlight the feasibility and safety of performing AI CS implantation via the TRA. TRA offers several advantages, including reduced access-site complications, shorter hemostasis time, and earlier ambulation, making it an attractive alternative to transfemoral access in selected patients. Recent device miniaturization has further expanded its feasibility.

BXB is compatible with a 6-Fr radial system up to a 7-mm stent size, although it can be expanded to 11 mm, allowing adequate luminal gain in most AI lesions. When severe calcification or tortuosity prevents safe deployment, early conversion to transfemoral access should be considered. Other balloon-expandable CS systems, such as LifeStream (Becton, Dickinson and Company), are also 6-Fr compatible and may be used in TRA-based strategies; however, LifeStream can only accommodate balloon diameters up to 6.0 mm or up to 7.0 mm × 26 mm within a 6-Fr system.

TRA should be avoided in patients with unfavorable aortic arch anatomy or a shaggy aorta due to the risk of embolization. With proper patient and device selection, TRA can serve as a minimally invasive and effective approach for AI CS implantation.

To prevent radial artery occlusion and ensure safe and smooth TRA EVT, preprocedural ultrasound assessment of the radial artery size is important, along with evaluation of the radial artery and aortic arch anatomy using magnetic resonance angiography or computed tomography [16]. Adequate anticoagulation with heparin should be maintained throughout the procedure to minimize the risk of thrombotic occlusion. When necessary, vasodilators and analgesics may be administered to achieve sufficient pain control. Furthermore, the application of hemostasis techniques that avoid excessive arterial compression may contribute to a lower incidence of radial artery occlusion.

## Conclusions

We report two successful cases of CS deployment for AI lesions using the TRA. With continued advancements in device technology, this strategy may become a promising alternative to conventional femoral access, offering a less invasive treatment option.

## Data Availability

The datasets used and/or analyzed during the current study are available from the corresponding author on reasonable request.

## Abbreviations

AI: Aortoiliac
TRA: Transradial approach
CS: Covered stent
EVT: Endovascular therapy
IVUS: Intravascular ultrasound
